# Bilastine: new insight into antihistamine treatment

**DOI:** 10.1186/s12948-015-0008-x

**Published:** 2015-04-15

**Authors:** Erminia Ridolo, Marcello Montagni, Laura Bonzano, Cristoforo Incorvaia, Giorgio Walter Canonica

**Affiliations:** Department of Clinical and Experimental Medicine, University of Parma, via Gramsci 14, Parma, 43126 Italy; Pulmonary Rehabilitation Unit, ICP Hospital, Milan, Italy; Allergy and Respiratory Diseases Clinic, DIMI, University of Genoa, IRCCS AOU San Martino-IST, Genoa, Italy

**Keywords:** Antihistamines, Bilastine, Allergic rhinitis, Chronic urticaria, Efficacy, Safety

## Abstract

Bilastine is a new second generation H1-antihistamine recently approved for the symptomatic treatment of allergic rhinitis (AR) and chronic urticaria (CU). Bilastine epitomizes the evolution of research on antihistamines concerning both efficacy and safety. In AR treatment, a number of large controlled clinical trials documented its efficacy, as assessed by improvement of all nasal and ocular symptoms and quality of life. These outcomes show that bilastine meets current EAACI/ARIA criteria for medications used in the treatment of AR. Also in CU, the review of the literature indicates that once-daily treatment with bilastine 20 mg was effective in managing symptoms and improving patient’s quality of life. Concerning safety and tolerability, the profile of bilastine is very similar to placebo and in particular the adverse effects on central nervous system are insignificant. The balance of efficacy and safety of bilastine is particularly helpful when dosages higher than standard are needed to control the symptoms, as frequently occurs in patients with urticaria, in whom antihistamines doses up to four times the standard dose may be administered.

## Introduction

Bilastine is a new second generation H1-antihistamine recently approved for the symptomatic treatment of allergic rhinitis (AR) and chronic urticaria (CU) in patients older than 12 years of age. AR and urticaria are very common clinical conditions that represent one of the most frequent reasons for a patient to visit their general practitioner or allergist. In the European countries 18% of population is suffering from AR [1] and in the United States AR affects a range of 10-30% of adults and up to 40% of children [2]. For urticaria, the life-time prevalence is approximately 20% for acute urticaria and 1.8% for CU [3]. These two clinical entities, though they have different and distinct characteristics, both cause a significant impairment of quality of life (QoL) and loss of productivity [4-6]. AR and urticaria respond to antihistamine treatment and current international guidelines recommend nonsedating second generation antihistamines as first line treatment for both [3,7,8].

The role of histamine in allergic inflammation is unequivocal. It exerts its biological effects acting on four distinct receptors, but among them the H1-receptor plays the most important role in allergic diseases. H1-antihistamines can control allergic inflammation by directly interfering with histamine action at H1 receptors [9]. Based on their ability to cross the blood–brain barrier, H1-antihistamines are classified into 2 groups: the first generation antihistamines that bind the H1-receptors on neurons in the central nervous system, causing sedation and impaired mental status, and the second-generation antihistamines (including bilastine) that usually cannot cross the blood–brain barrier and thus have fewer sedative effects. The long duration of action, efficacy, low-sedation impact and low-performance impairment of second generation antihistamines, and particularly of bilastine, make it a potentially attractive therapeutic option for allergists.

This review focuses on the clinical characteristics of bilastine and the evidence of its efficacy in the treatment of AR and urticaria.

## Review

### Pharmacodynamic and pharmacokinetic properties

From a molecular point of view, bilastine is 2-[4-(2-(4-(1-(2- ethoxyethyl)-1Hbenzimidazol-2-yl) piperidin-1-yl) ethyl) phenyl]-2-methyl propionic acid. It belongs to piperidine derivatives and is not structurally derived from any other currently available antihistamines. Bilastine exerts a potent and specific H1-antihistamine activity.

Bilastine is an H1 receptor inverse agonist, like other antihistamines already available. Preclinical in vitro studies showed that bilastine has high specificity for H1-receptors while has negligible affinity for 30 other receptors (serotonin, bradykinin, leukotriene-D4, muscarinic M3-receptors, α1-adrenoceptors, β2-adrenoceptors, and H2- and H3-histamine receptors) [10]. The affinity for the H1 receptor is 3 and 6 times higher than for cetirizine and fexofenadine, respectively. The results of *in vivo* preclinical studies confirmed those obtained from *in vitro* experiments, as in rats bilastine has demonstrated to reduce histamine-stimulated smooth muscular contraction, bronchospasms, endothelial permeability, and microvascular extravasation, providing evidence to possess antiallergic properties, with similar potency to cetirizine and superior potency to fexofenadine [11].

In vitro data have shown that bilastine also exerts anti-inflammatory activity by inhibiting the release of histamine, IL-4 and tumor necrosis factor (TNF)-α from human mast cells and granulocytes [12].

The drug is rapidly absorbed after oral administration with a time to maximum plasma concentration of around 1 hour following administration [13]. The mean value of bilastine oral bioavailability was found to be about 60% [14]. The maximum plasma concentration (220 ng/mL) of bilastine 20 mg was detected 1.3 hours after administration, the half time was 14.5 hours, and plasma protein binding was 84–90% [13,14]. Bilastine does not undergo any significant hepatic metabolism and approximately 95% is excreted intact in faeces (67%) or in urine (33%); bilastine is a substrate for P-glycoprotein which limits its passage across the blood–brain barrier [15], and not clinically relevant interactions have been reported to date. The mean elimination half-life calculated in healthy volunteers was 14.5 h and the apparent total plasma clearance is 18.1 L/h [16,17]. Bilastine is not a substrate of CYP450 family [18].

### Efficacy of bilastine

#### Wheal and flare inhibition

A phase 1, double-blind, randomised, placebo-controlled, single oral dose, cross-over study compared the antihistaminic effects of bilastine, cetirizine and placebo against histamine-induced wheal and flare responses, over periods of 24 h, in 21 healthy male volunteers [19]. In that trial, volunteers were randomised to receive single oral doses of bilastine 20 or 50 mg, cetirizine 10 mg or placebo before provocation of wheal and flare responses to 100 mg/ml histamine by skin prick 1.5, 4, 8, 12 and 24 h later. The authors found no significant differences between overall inhibitions of wheal or flare by bilastine 20 mg and cetirizine10 mg but bilastine was faster in onset of action than cetirizine, inhibition of wheal and flare at 1.5 h being 89 ± 3 versus 44 ± 14% (P = 0.011) and 85 ± 4 versus 45 ± 14% (P = 0.016), respectively. At 1.5 h, both wheals and flares were inhibited by 70% in 11/12 volunteers taking bilastine and 3/11 taking cetirizine (P = 0.003). There were no significant differences between the drugs at later times.

### Bilastine efficacy in allergic rhinitis

Efficacy of bilastine was well recognized in both seasonal and perennial allergic rhinitis. The Vienna Challenge Chamber is an established standardized method for the controlled exposure of patients to defined allergens, that is used to make comparisons between different antihistamines [20,21]. Using this method, a double blind, randomized, placebo controlled, balanced four-treatment, four-period crossover phase II study was conducted in patients suffering from seasonal AR (SAR) in order to compare the efficacy of bilastine, cetirizine and fexofenadine to relieve symptoms [22]. The study was conducted in adult patients with confirmed allergy to grass pollen, outside the pollen season while they were asymptomatic. Total Nasal Symptoms Score (TNSS) was used to compare the effect of a single dose of bilastine 20 mg, cetirizine 10 mg, fexofenadine 120 mg and placebo administered two hours after the start of the challenge. During the first four hours after administration, all treatment were significantly more effective than placebo in reducing TNSS (p < 0.001), without significant difference between the three antihistamines. Moreover, bilastine at 20 mg was as effective as cetirizine 10 mg and fexofenadine 120 mg in terms of onset of action and in reducing eye symptoms 1 h after the intake. Bilastine was still effective 26 hours after the intake, confirming the prolonged duration of action.

The efficacy of bilastine in patients with SAR has also been evaluated in two double-blind, placebo-controlled studies, with the same design, evaluating parameters for efficacy and safety, including assessment of QoL in the first one, comparing once daily bilastine 20 mg with placebo, desloratadine 5 mg and cetirizine 10 mg over two weeks [23,24]. Details of the two studies are provided in table 1. These two studies enrolled a total of 1404 patients, aged between 12 and 70 years, with documented SAR due to pollen allergens. In both studies primary outcome measure of TSS was significantly reduced in the bilastine group significantly more than in the placebo group and to a similar extent as in the active comparator group (table 1). Also, bilastine improved QoL, measured by rhinoconjunctivitis quality of life questionnaire (RQLQ), to a similar extent than desloratadine; bilastine 20 mg significantly improved total RQLQ score and most RQLQ single domains respect to placebo.

**Table 1:** **Double blind randomized trials in seasonal AR**

**Study**	**Patients N.**	**Duration**	**Treatment**	**Efficacy**	**Safety vs active comparator**
Kuna P et al. [24]	683	14 days	Bilastine 20 mg Cetirizine 10 mg Placebo	The mean TSS-AUC_0_14_ days (score_day) was reduced in bilastine and cetirizine groups to a similar and significantly greater extent, compared with placebo (P < 0.001). Bilastine and cetirizine were comparable and significantly superior to placebo for all secondary outcomes	Significantly fewer patients in the bilastine-treated group experienced somnolence (P < 0.001) and fatigue (P = 0.02) than patients in the cetirizine-treated group.
Bachert C et al. [23]	721	14 days	Bilastine 20 mg Desloratadine 5 mg Placebo	The AUC of TSS was decreased to a significantly greater extent in the bilastine group compared with placebo group (P < 0.001). Total RQLQ score was significantly reduced from baseline by a value of 1.6 (1.2; 1.8–1.4) in the bilastine treated group compared with a value of 1.3 (1.3; 1.5–1.1) in the placebo-treated group (P < 0.005)	Safety profile of bilastine and desloratadine were comparable to placebo.

TSS-AUC_0_14_: area under the curve (AUC) of the reflective total symptoms score (TSS) from day 0 (D0) today 14; RQLQ: rhinoconjunctivitis quality of life questionnaire.

A multicenter, randomized, placebo-controlled, double-blind, parallel-group study was conducted in 650 patients with symptomatic persistent AR (PAR) [25]. The authors found no significant differences in efficacy outcomes between active treatments and placebo in the overall population, over 4 weeks of treatment, but in the post-hoc analysis bilastine 20 mg was more effective than placebo and as effective as cetirizine 10 mg the population of patients from Europe and Argentina, while the difference remained not statistically significant in patients from South Africa. The lack of efficacy through the whole group was likely to be due to the group great variability of symptom scores reported in different countries, particularly in South Africa [26].

Davila et al. and Bartra et al. analyzed the data about the effect of bilastine upon nasal obstruction and ocular symptoms from 7 phase II and phase III 2–4 weeks duration clinical trials [27,28]. Mean change from baseline in nasal obstruction symptoms score after two weeks of treatment was −0.66 with bilastine 20 mg and −0.57 with placebo (p < 0.001), active comparators (cetirizine 10 mg and desloratadine 5 mg) induced a reduction of −0.67 points (p < 0.001 vs placebo; not statistically different vs bilastine) [27]. Similarly, bilastine was more effective in ocular symptoms relief than placebo and as effective as active comparators [28].

### Bilastine efficacy in urticaria

A multi-centre, double-blind, randomized, placebo-controlled study compared the efficacy and safety of bilastine 20 mg vs levocetirizine 5 mg for the treatment of chronic idiopathic urticaria in 525 adult patients [29]. The TSS was reduced progressively by all treatments from baseline over a period of 28 days treatment, with significant differences noted between bilastine 20 mg and levocetirizine 5 mg-treated groups vs placebo-treated group from day 2 onward over the entire treatment period . The mean change from baseline in the patients’ reflective daily TSS over the 28-day treatment period, that was the primary efficacy measure, was significantly greater for bilastine 20 mg and levocetirizine 5 mg treated groups compared with placebo-treated group (P < 0.001 for bilastine and levocetirizine vs placebo), but not significantly different between the active treatment groups.

Cold urticaria is a quite uncommon form of inducible urticaria, characterized by pruritic wheals and/or angioedema due to cutaneous mast cell activation and release of pro-inflammatory mediators after cold exposure [30]. Reduction of symptoms in many patients with cold urticaria requires high dosing with antihistamines, up to four times the daily recommended dose [31,32]. Krause et al. assessed the effects of the standard 20 mg dose and up-dosing to 40 and 80 mg of bilastine in reducing the symptoms of CU and inflammatory mediator release following cold challenge in a randomized, crossover, double-blind, placebo-controlled 12-week study [33]. In this study patients suffering from cold urticaria, confirmed by a specific provocation test, received placebo, 20, 40 or 80 mg of bilastine daily each for 7 days with 14-day washout periods. Bilastine was effective already at routine doses: in patients receiving 20 mg, the critical temperature threshold (CCT, the highest temperature that produces a positive wheal response) was significantly different from placebo (the median CCT value was 6°C in bilastine group and 18°C in placebo group), P < 0.0001), as well as the number of patients that became symptom free (P = 0.044). The up-dosing was beneficial, since the median CTT with bilastine 80 mg was significantly lower than that of 20 mg (P = 0.003) and 40 mg (P = 0.04). Moreover, inflammatory mediators were significantly reduce by bilastine 80 mg.

### Safety of bilastine

Tolerability data from the four phase III trials of 2–4 weeks duration are summarized in table 2. In these studies, bilastine was well tolerated and the majority of the adverse events described were either mild or moderate, while no serious adverse events or death were reported; moreover, there were no clinically significant changes in any laboratory tests, ECGs, heart rate, or systolic and diastolic blood pressure, in patients treated with bilastine 20 mg. The most common adverse effects were headache, somnolence and fatigue that were reported less frequently than in patients receiving cetirizine at 10 mg once daily. The frequency of these adverse effects in patients with SAR were comparable with that of desloratadine. The first generation antihistamines cross the blood–brain barrier and bind the H1-receptors on postsynaptic neurons membranes in the central nervous system, causing sedation and impaired mental status, while the second-generation antihistamines usually do not cross the blood–brain barrier and have fewer sedative effects. Bilastine 20 mg histamine H1-receptor occupancy had been evaluated by positron emission tomography (PET) in healthy subjects, confirming that bilastine has objective and PET criteria to be defined as a non-sedating antihistamine [34]. Moreover, bilastine demonstrated to produce only very little or even no performance impairment. In a crossover, randomized, double-blind, placebo-controlled study 20 healthy volunteers received repeated doses of bilastine 20, 40, or 80 mg and first generation antihistamine hydroxyzine 25 mg on 7 consecutive days [35]. Bilastine in doses up to 40 mg did not produce psychomotor impairment as compared with placebo, even if 40 mg produced subjective report of sedation, and an objective impairment was only evident at bilastine doses of 80 mg. Similarly, bilastine did not produce any driving impairment after single and repeated doses up to 40 mg [36]. At the therapeutic dose of 20 mg the concomitant administration of bilastine and alcohol does not produce greater central nervous system (CNS) depressant effects than alcohol alone, while objective impairment induced bilastine 80 mg + alcohol (0.8 g/Kg) was of similar magnitude to that induced by hydroxyzine 25 mg + alcohol [37]. Also cardiac safety was confirmed at therapeutic and supra-therapeutic doses. Bilastine administration at 20 mg and 100 mg had no clinically significant impact on QTc. Concomitant administration of ketoconazole and bilastine 20 mg induced a clinically relevant increase in QTc but this result was most likely related to the cardiac effect of ketoconazole [38]. Moreover, bilastine, at therapeutic and supratherapeutic dosages (up to 100 mg), did not induce any effects on T-wave morphology [39].

**Table 2:** **AEs in patients receiving bilastine 20 mg in clinical trials**

**Study**	**Patients N. Duration**	**Disease**	**AEs in bilastine-treated group**
Kuna P et al. [24]	683	SAR	Any 24.7%
	14 days		
			Headache 10.6%
			Somnolence 1.8%
			Fatigue 0.4%
			Dyspnoea 0.9%
Bachert C et a. [23]	721	SAR	Any 28.3%
	14 days		Headache 12.0%
			Somnolence 3.9%
			Fatigue 2.6%
Sastre J et al. [25]	650	PAR	Any 23.4%
	4 weeks		
			Headache 10.7%
			Somnolence 13.7%
Zuberbiert T et al. [29]	525	CIU	Any 30.1%
	28 days		Headache 12.1%
			Somnolence 5.8%
			Fatigue 2.9%

SAR: seasonal allergic rhinitis; PAR: persistent allergic rhinitis, CIU: chronic idiopatic urticarial, AEs: adverse event.

## Conclusions

Bilastine epitomizes the evolution of research on antihistamines concerning both efficacy and safety [40]. In AR treatment, its efficacy has been documented in several large controlled clinical trials [26]. Bousquet et al. in 2012 reviewed the available literature and found that bilastine 20 mg once daily improved all nasal and ocular symptoms of AR and improved quality of life, an important outcome in allergic diseases. Therefore, the authors concluded that bilastine meets current EAACI/ARIA criteria for medications used in the treatment of AR [41]. In a comparable review of the medical literature on the effectiveness of bilastine in urticarial syndromes, either spontaneous or inducible, Jauregui et al. concluded that once-daily treatment with bilastine 20 mg was effective in managing symptoms and improving patient’s quality of life in chronic urticaria [42]. Concerning safety and tolerability, the profile of bilastine is very similar to placebo in all Phase I, II and III clinical trials. Differently from most antihistamines, bilastine does not increase the CNS depressant effect of lorazepam and, unlike other second-generation antihistamines such as cetirizine, does not increase alcohol effects on the CNS [43]. The balance of efficacy and safety of bilastine is particularly helpful when dosages higher than standard are needed to control the symptoms. This is of particular importance when the doses are much higher, as frequently occurs in patients with urticaria, where antihistamines doses up to four times the standard dose are administered, patient safety being a key requirement when choosing a specific antihistamine [44].
